# Translation, cultural adaptation and validation of the facial disability index into Brazilian Portuguese^☆^

**DOI:** 10.1016/j.bjorl.2019.04.003

**Published:** 2019-05-18

**Authors:** Agnaldo J. Graciano, Marcela M. Bonin, Marion R. Mory, Adriana Tessitore, Jorge R. Paschoal, Carlos T. Chone

**Affiliations:** a Universidade Estadual de Campinas (Unicamp), Faculdade de Ciência Médicas, Departamento de Otorrinolaringologia, Campinas, SP, Brazil; b Hospital de Clínicas, Serviço de Fonoaudiologia, Ambulatório de Paralisia Facial, Campinas, SP, Brazil

**Keywords:** Facial nerve, Facial paralysis, Complications, Assessment of disability, Patient Health Questionnaire, Nervo facial, Paralisia facial, Complicações, Avaliação da deficiência, Questionário de saúde do paciente

## Abstract

**Introduction:**

Facial paralysis may occur due to a variety of causes. It is associated to the impairment of some basic daily activities such as eating, drinking, speaking and social communication, which affects the quality of life of these patients. The facial disability index is a short form auto reported outcome questionnaire used to assess patient with facial paralysis. It has been validated and proved to be superior to other general health related quality of life questionnaires.

**Objective:**

We aim to do the cultural adaptation and validate the facial disability index into Brazilian Portuguese.

**Methods:**

Translation and cultural-adaptation following the stages recommended by the International Society of Pharmacoeconomics Outcomes Research task force. The questionnaire was administered to 100 patients for evaluation of reliability and validation.

**Results:**

The reliability of the Portuguese version of the facial disability index was found to be adequate, with a Cronbach’s alfa coefficient of 0.73 for the complete scale. Intra-class correlation was 0.79 (95% CI: 0.71–0.85) and 0.85 (95% CI: 0.78–0.89) for the physical and social well-being subscales. There was a significant correlation between the social well-being subscale of the Portuguese version of the facial disability index and the social function and mental health components of the SF-36. There was also a correlation between the facial disability index and the degree of facial dysfunction according to the House–Brackmann global scale.

**Conclusion:**

This adapted version of the facial disability index provides a valid and reliable instrument to assess the physical and psychosocial impact of facial nerve dysfunction in Brazilian-speaking patients.

## Resumo

**Introdução:**

Paralisia facial periférica pode ocorrer devido a uma grande variedade de causas e está associada ao comprometimento de atividades diárias básicas, como comer, beber, falar e comunicação social, afeta a qualidade de vida dos pacientes. O *facial disability index* é um questionário autoaplicado desenvolvido especialmente para avaliar o impacto da disfunção facial sobre aspectos físicos e psicossociais dos pacientes. Embora tenha sido validado e se mostrado superior a outros questionários, ainda não fora submetido à adaptação transcultural e validação para a língua portuguesa.

**Objetivo:**

Realizar a tradução, adaptação cultural e validação do *facial disability index* para o português falado no Brasil.

**Método:**

A tradução e adaptação cultural do *facial disability index* foram realizadas em diferentes estágios conforme recomendações internacionais para adaptação de medidas de resultados. Para a verificação de confiabilidade e validação da versão em português, o facial disability index foi aplicado em 100 pacientes na forma de teste/reteste.

**Resultados:**

A confiabilidade da versão em português do facial disability index foi considerada adequada, com coeficiente alfa de Cronbach de 0,73. A avaliação de correlação intraclasse foi de 0,79 (95% IC 0,71–0,85), e de 0,85 (95% IC 0,78–0,89) para as subescalas física e de bem-estar social, respectivamente. Houve uma correlação significativa entre a escala de bem-estar social da versão em português do *facial disability index* e as dimensões de função social e saúde mental do questionário geral de qualidade de vida *Short Form*-36. Também foi demonstrada uma correlação entre a versão em português do *facial disability index* e o grau de disfunção facial de acordo com a escala global de House-Brackmann.

**Conclusão:**

A versão adaptada do *facial disability index* para o português falado no Brasil se apresenta como um instrumento válido e confiável para avaliação do impacto da disfunção facial sobre aspectos físicos e psicossociais dos pacientes.

## Introduction

Facial paralysis can result from a wide variety of clinical, traumatic, and iatrogenic causes.^1^, ^2^

Dysfunction of orofacial motricity resulting from these conditions can lead to different degrees of basic function alterations such as eating, drinking and speaking, in addition to expected impairment in facial mimetics and its consequences in the capacity of interaction and social expression.^3^ Consequently, facial disability reflects the overall perception of patients about their health and quality of life. One of the main obstacles to the subjective evaluation of quality of life in patients with facial paralysis is the limitation of general questionnaires to discriminate the specific difficulties faced by these individuals. Although some questionnaires were developed to assess patients with facial paralysis, few have been adequately validated.^4^ The Facial Disability Index (FDI) is a self-reported questionnaire that assesses quality of life aspects related to the physical and psychosocial limitations resulting from orofacial motor alterations. It has been shown to be valid and more specific than other general quality of life assessment tools for patients with facial paralysis.^5^ Although it has been used in several studies and adapted for other languages,^6^, ^7^, ^8^ the FDI has not yet undergone cultural adaptation and validation into Portuguese. Therefore, the aim of this study was to perform the translation, cultural adaptation and validation of the Facial Disability Index into Brazilian Portuguese.

## Patients and methods

This study was approved by the institution's Research Ethics Committee (CAAE: 49967415.0.0000.5404) and all participants gave their written free and informed consent to participate in the study, according to the Declaration of Helsinki.

The FDI questionnaire consists of 10 items, separated into physical and social well-being subscales, each with 5 items quantified on a 6-point scale and transformed into a score of up to 100 points that would indicate unaltered physical and social well-being functions. The physical subscale investigates problems when eating, drinking, speaking, performing oral hygiene, and ocular symptoms, such as tearing or ocular dryness. The social welfare subscale investigates aspects related to anxiety, irritability and social interaction.

The authorization of the American Physical Therapy Association, which holds the publication rights, was obtained for the translation and cultural adaptation of the FDI. The technique used followed the international recommendations for translation and cultural adaptation of measures of self-reported results,^9^ including the following steps:

1) Initial translation based on the original FDI in American English into Portuguese, carried out by two independent qualified translators, whose first language was Portuguese.
2) The reconciliation of the obtained versions were evaluated by two researchers involved in the project and compared for differences and preparation of a single initial version of the Portuguese translation.
3) Back-translation: in this phase the initial Portuguese version was back-translated into English by two other translators who are native speakers of American English and who were unaware of the original questionnaire.
4) Review of back-translations: comparing them with the original questionnaire and the reconciliation version to determine if they showed literal results different from the original tool, or similar ones, maintaining the concept of the questionnaire. At this phase, the developer of the original questionnaire was contacted to clarify doubts about the concept of some items.
5) A second version of the FDI was carried out by a committee involved in the study based on the harmonization of the comparisons of the Portuguese translations and the back-translations.
6) Cognitive research: At this phase, the test version of the questionnaire was applied to 20 patients to verify the need for the use of alternative words, interpretability, comprehensibility, and cultural relevance of the translation. The questionnaire was self-administered by the patients and after answering it, the researchers asked them to record the understanding of each item on an analogue scale, graduating it from 1 to 10 (from the most difficult to understand to the easiest). The clarity index of each question was obtained by the mean of the values over the number of patients, and scores lower than 0.4 would represent misinterpreted question wordings; between 0.5 and 0.7, unclear wordings and, above 0.8, clear wordings, as proposed by Tavares et al.^10^
7) The committee involved in the translation evaluated the unfolding of the cognitive research and compared the versions obtained in the previous steps to determine discrepancies of the original intended meaning, creating the final version of the FDI in Portuguese (Pt-FDI).

For the validation of Pt-FDI, the final version of the questionnaire was applied using the test-retest format in a group of 100 outpatients diagnosed with peripheral facial paralysis.

### Inclusion criteria

Adult patients over 18 years of age with a diagnosis of peripheral facial paralysis.

### Exclusion criteria

Illiterate patients, or significant cognitive limitations.

Exploratory data analysis was performed through summary measures (mean, standard deviation, minimum, median, maximum values, frequency and percentage).

The internal consistency of the Portuguese version of the FDI was evaluated through Factorial Analysis and Cronbach's alpha for each of the subscales.

The agreement between the FDI application times (Test–Retest) was performed using the Intraclass Correlation Coefficient (ICC).

To analyze the construct validity of the Portuguese version of the FDI questionnaire, the obtained results were compared with the degree of facial dysfunction according to the House–Brackmann scale for facial paralysis.^11^

The comparison of the FDI between the degrees of paralysis was performed using the Kruskal–Wallis test, followed by Dunn test for multiple comparisons.

The results of the Portuguese FDI questionnaire subscales were compared with the functional capacity, social aspects and mental health data of the SF-36 (Medical Outcome Study 36 – Short Form Health Survey Item) translated and validated into Portuguese^12^ and answered by 50 patients). The correlation of the FDI with SF-36 was assessed using Spearman's coefficient. The level of significance was set at 5%.

## Results

The validation of the Portuguese version of the FDI self-reported questionnaire was carried out between March 2015 and July 2017, with the participation of 100 adult patients aged 18–85 years (mean of 48.5 years) with facial paralysis of different etiologies (58% due to surgical sequela, 42% due to other causes), treated at the Facial Paralysis Outpatient Clinic of a tertiary institution.

The Portuguese version of the FDI was considered easy to understand by patients, with all items showing an understanding score above 0.96 for all questions.

The final version of the FDI in Portuguese has been translated literally for most of the items, considering the objectivity of the original questionnaire and the possibility of preserving the meaning of the questions (Table 1). A conceptual and cultural adaptation was made to Item 3: “*How much difficulty did you have saying specific sounds while speaking*?”, which has been adapted to: “*How difficult was it for you to speak*?”; and also to Item 9: “*How often did you wake up early or wake up several times during your nighttime sleep?”*, which was adapted to: “*How often did you lose your sleep or woke up several times during the night?”.* These changes were considered necessary by the group involved in the cultural adaptation of the questionnaire, aiming to facilitate the understanding of the wordings in Portuguese.

**Table 1:** Brazilian-Portuguese Version of the Facial Disability Index.

**Índice de Disfunção Facial**	
*Por favor, escolha a resposta mais apropriada para as seguintes questões relacionadas a problemas associados com a função de seus músculos faciais*	
**Para cada pergunta, considere sua função facial durante o último mês:**	

**Função Física**	
*1. Quanta dificuldade você teve para manter a comida na boca, mover a comida dentro da boca ou por ficar com a comida parada na bochecha enquanto comia?*	
Geralmente comi com	
5 = Nenhuma dificuldade	
4 = Um pouco de dificuldade	
3 = Alguma dificuldade	
2 = Muita dificuldade	
1 = Geralmente não comi por motivo de saúde	
0 = Geralmente não comi por outras razões	

*2. Quanta dificuldade você teve para beber com copo?*	
Geralmente bebi com	
5 = Nenhuma dificuldade	
4 = Um pouco de dificuldade	
3 = Alguma dificuldade	
2 = Muita dificuldade	
1 = Geralmente não bebi por motivos de saúde	
0 = Geralmente não bebi por outras razões	

*3. Quanta dificuldade você teve para falar?*	
Geralmente falei com	
5 = Nenhuma dificuldade	
4 = Um pouco de dificuldade	
3 = Alguma dificuldade	
2 = Muita dificuldade	
1 = Geralmente não falei por motivo de doença	
0 = Geralmente não falei por outras razões	

*4. Quanta dificuldade você teve por ficar com seu olho lacrimejando excessivamente ou por ficar com o olho ressecado?*	
Geralmente tive	
5 = Nenhuma dificuldade	
4 = Um pouco de dificuldade	
3 = Alguma dificuldade	
2 = Muita dificuldade	
1 = Geralmente não observei por motivo de saúde	
0 = Geralmente não observei por outros motivos	

*5. Quanta dificuldade você teve para escovar os dentes ou enxaguar a boca?*	
Geralmente tive	
5 = Nenhuma dificuldade	
4 = Um pouco de dificuldade	
3 = Alguma dificuldade	
2 = Muita dificuldade	
1 = Geralmente não escovei os dentes ou enxaguei a boca por motivo de saúde	
0 = Geralmente não escovei os dentes ou enxaguei a boca por outras razões	

**Função Bem-estar Social**	
*6. Com que frequência você se sentiu calmo e tranquilo?*	
6 = O tempo todo	
5 = A maior parte do tempo	
4 = Uma boa parte do tempo	
3 = Algumas vezes	
2 = Poucas vezes	
1 = Nenhuma vez	

*7. Com que frequência você se isolou das pessoas ao seu redor?*	
1 = O tempo todo	
2 = A maior parte do tempo	
3 = Uma boa parte do tempo	
4 = Algumas vezes	
5 = Poucas vezes	
6 = Nenhuma vez	

*8. Com que frequência você ficou irritado com as pessoas ao seu redor?*	
1 = O tempo todo	
2 = A maior parte do tempo	
3 = Uma boa parte do tempo	
4 = Algumas vezes	
5 = Poucas vezes	
6 = Nenhuma vez	

*9. Com que frequência você perdeu o sono ou acordou várias vezes durante a noite?*	
1 = Todas as noites	
2 = A maioria das noites	
3 = Várias noites	
4 = Algumas noites	
5 = Poucas noites	
6 = Nenhuma noite	

*10. Com que frequência sua função facial lhe impediu de sair para comer, fazer compras ou participar de atividades familiares ou sociais?*	
1 = O tempo todo	
2 = A maior parte do tempo	
3 = Uma boa parte do tempo	
4 = Algumas vezes	
5 = Poucas vezes	
6 = Nenhuma vez	

**Escore Função Física** Pontuação (questões 1-5) – *N* × 25 = ______	**Escore Função Social/Bem-Estar** Pontuação (questões 6-10) – *N* × 20 = ______
*N*	*N*

Escore FDI total = escore função física + escore função social/bem-estar = ________	
200	

The internal consistency of the Portuguese version of the FDI was considered adequate, with a Cronbach's alpha coefficient of 0.73 (Table 2).

**Table 2:** Evaluation of reliability and internal consistency of the Pt-FDI.

Item	Alpha if Item was excluded
Q1	0.6314
Q2	0.7042
Q3	0.6509
Q4	0.7713
Q5	0.7114
Q6	0.7367
Q7	0.7387
Q8	0.7022
Q9	0.7367
Q10	0.6893
Global	0.735

Q, Question.

The reliability determined in the test/retest showed an Intraclass Correlation Coefficient (ICC) of 0.79 for the physical subscale, 0.85 for the social well-being subscale, and 0.88 for the entire questionnaire (Table 3).

**Table 3:** Evaluation of the Pt-FDI Test-Retest agreement (*n* = 100).

Variable	Mean	SD	ICC	95%CI
FF – Q1	66.2	17.7	0.79	0.71–0.86
FF – Q2	66.6	19.1		
FBES – Q1	69.4	21.6	0.85	0.79–0.90
FBES – Q2	71.6	22.7		
Total – Q1	0.68	0.17	0.88	0.82–0.92
Total – Q2	0.69	0.23		

Pt-FDI, FDI in Portuguese; SD, standard deviation; ICC, Intraclass Correlation Coefficient; CI, confidence Interval.

Comparison of FDI subscales with components of the SF-36 demonstrated a correlation between the FDI social well-being scale and the mental health measures, social aspects, and functional capacity of the SF-36. On the other hand, the FDI physical function scale did not correlate with these measures (Table 4), as observed in the original FDI study.^5^

**Table 4:** Bivariate correlation of Pearson and Spearman between the Pt-FDI questionnaire and the SF-36.

Bivariate correlation	Pearson's corr	Pearson's 95% CI	Spearman's rank	Spearmen's 95% CI	*p* value
MH SF36/SWB–FDI	0.59	0.38 to 0.75	0.57	0.34 to 0.73	<0.0001
PA SF36/SWB–FDI			0.52	0.28 to 0.70	<0.0001
FC SF36/SWB–FDI			0.27	−0.005 to 0.51	0.05
PA SF36/PF–FDI			0.24	−0.039 to 0.48	0.09
FC SF36/PF–FDI			0.21	−0.06 to 0.46	0.13
MH SF36/PF–FDI	0.12	−0.16 to 0.38			0.4

Pt-FDI, *Facial Disability Index* in Portuguese; SF, Short Form Questionnaire; Corr., Correlation; CI, Confidence Interval; MH, Mental Health; SWB, Social Well-Being; PA, Physical Aspects; PF, Physical Function; FC, Functional Capacity.

The FDI physical subscale correlated with the degree of facial dysfunction assessed by the HB scale, with significant differences in the physical function means among individuals with mild facial dysfunction (House–Brackmann-2), moderate (HB-3), moderately severe (HB-4), or severe/total facial dysfunction (HB-5/6) (*p* < 0.0001). In the paired comparison between the degrees of dysfunction, it was observed that the means of the physical function subscale correlated with the difference between mild dysfunctions compared to moderate or severe dysfunctions. The mean scores of the social well-being subscale were also correlated with the degree of facial dysfunction (*p* = 0.0023); being more evident when comparing individuals with mild dysfunction (HB-2) *versus* moderately severe (HB-4) (*p* = 0.02) or severe (HB-5/6) dysfunction (*p* = 0.0012) (Table 5).

**Table 5:** Correlation between the Pt-FDI subscales and the degree of facial dysfunction according to the House–Brackmann scale (Kruskal–Wallis test/Dunn test).

Variable	HB grade	*N*	Mean	SD	*p* value
Physical function	2	18	87.78	11.14	<0.001
	3	24	68.13	15.24	
	4	43	58.02	14.89	
	5/6	15	60	14.02	
	2 *vs*. 3		19.65		0.0001
	2 *vs*. 4		29.75		<0.0001
	2 *vs*. 5/6		27.78		<0.0001
	3 *vs*. 4		10.1		0.03
	3 *vs*. 5/6		8.125		0.31
	4 *vs*. 5/6		−1.98		0.96

Social well-being function	2		83.78	18.38	0.002
	3		70.29	21.03	
	4		67.19	20.31	
	5/6		56.27	22.4	
	2 *vs*. 3		13.49		0.15
	2 *vs*. 4		16.59		0.02
	2 *vs*. 5/6		27.51		0.001
	3 *vs*. 4		3.1		0.93
	3 *vs*. 5/5		14.02		0.16
	4 *vs*. 5/6		10.92		0.29

Pt-FDI, Facial Disability Index in Portuguese; HB, House–Brackmann; *N*, number of patients; SD, standard deviation.

## Discussion

This study demonstrates that the Portuguese version of the FDI questionnaire is a valid and reliable tool for the evaluation of patients with facial paralysis. An overall Cronbach's alpha coefficient of the questionnaire > 0.7 was observed, and the intraclass correlation coefficient was around 0.8 for both subscales. These results are similar to those observed by Gonzales-Cardero et al.^8^ who observed a Cronbach's alpha coefficient of 0.8 for the Spanish version of the FDI. It is possible that the relatively lower alpha value observed in the Spanish study may have been influenced by the use of a larger sample, including patients with facial paralysis of different etiologies, whereas this study evaluated only patients submitted to parotidectomy, who generally have a lower degree of facial paralysis and no involvement of all branches of the facial nerve.

We observed a significant correlation between the measures of the social well-being subscale of the Portuguese version of the FDI with the components of mental health and social aspects of the SF-36 global quality of life questionnaire. This correlation has also been demonstrated in the original FDI development work described by Van Swearingen and Brach,^5^ suggesting the effectiveness of the FDI to evaluate the impact of facial paralysis on psychosocial aspects.

As expected from the literature data, a correlation between the functional capacity measured by the SF-36 questionnaire and the FDI physical scale measures was not observed. Because it is an overall quality of life questionnaire, the SF-36 evaluates functional aspects that are not directly related to the physical incapacity caused by facial paralysis. Therefore, FDI would be more specific for the evaluation of physical alterations related to facial neuromuscular dysfunction.^5^

We observed that the mean values of the physical subscale measurements of the Portuguese version of the FDI were significantly related to the degree of facial paralysis according to the House–Brackmann scale, confirming the validity of the FDI to assess the association between facial dysfunction and physical disability. The correlation between the physical scale of the FDI and the degree of facial dysfunction was also demonstrated by Pavese et al.^6^ who used the Sunnybrook system for the classification of facial paralysis. Recently, Pratz-Golcer et al.^13^ demonstrated that the association between the physical scale of the FDI and the degree of facial paralysis persists throughout patient clinical evolution.

Considering the lack of tools translated and validated into Portuguese to evaluate results in patients with facial paralysis, we observed that this version of FDI is a useful tool in daily clinical practice. It is simple and easy to understand, allowing its multidisciplinary use, and can be applied in the follow-up of specific treatments or to evaluate the impact of surgical procedures on facial function.

## Conclusion

The Brazilian-Portuguese version of the FDI questionnaire is a valid tool for the evaluation of patients with facial paralysis.

## Conflicts of interest

The authors declare no conflicts of interest.
