# Supplementary material for: Translation, cultural adaptation and validation of the facial disability index into Brazilian Portuguese
Source: Braz J Otorhinolaryngol. 2019 May 18;86(5):602–8. doi: 10.1016/j.bjorl.2019.04.003 (PMC9422608; doi:10.1016/j.bjorl.2019.04.003)
Supplement: Supplementary file 1 [file mmc1.pdf]

PARECER CONSUBSTANCIADO DO CEP

DADOS DO PROJETO DE PESQUISA

Título da Pesquisa: Adaptação cultural e validação do Índice de Incapacidade Facial para português falado no Brasil

Pesquisador: Agnaldo José Graciano

Área Temática:

Versão: 1

CAAE: 49967415.0.0000.5404

Instituição Proponente: Hospital de Clínicas da UNICAMP

Patrocinador Principal: Financiamento Próprio

DADOS DO PARECER

Número do Parecer: 1.304.170

Apresentação do Projeto:

Adequada. Paralisia facial pode ocorrer devido a uma grande variedade de causas clínicas, traumáticas, e iatrogênicas, como complicações ou sequelas cirúrgicas. A disfunção da motricidade facial resultante dessas condições pode levar a diferentes graus de alterações de funções básicas como comer, beber, falar, além de esperado prejuízo na mímica facial e suas consequências na capacidade de interação e expressão social. Consequentemente, a incapacidade facial se reflete na percepção geral do paciente sobre sua saúde e qualidade de vida. Um dos principais obstáculos para se avaliar subjetivamente a qualidade de vida de pacientes com paralisia facial é a limitação que questionários gerais têm para discriminar as dificuldades específicas enfrentadas por estes indivíduos. Desenvolvido para superar estas limitações, o Facial Disability Index (FDI) é um questionário que acessa aspectos da qualidade de vida relacionados às limitações físicas e psicossociais decorrentes de alterações da motricidade facial, e que se mostrou válido e mais específico que outras ferramentas gerais de avaliação de qualidade de vida4. Embora tenha sido utilizado em vários estudos e adaptado para outras línguas, o FDI ainda não foi submetido à adaptação cultural e validação para o português. Portanto, o objetivo deste estudo é realizar a adaptação cultural e validação do FDI para o português falado no Brasil.

Endereço: Rua Tessália Vieira de Camargo, 126
Bairro: Barão Geraldo **CEP:** 13.083-887
UF: SP **Município:** CAMPINAS
Telefone: (19)3521-8936 **Fax:** (19)3521-7187 **E-mail:** cep@fcm.unicamp.br

Objetivo da Pesquisa:

Adaptação e validação do questionário “Facial Disability Index”, realizando a tradução para o português com as adequadas adaptações transculturais e linguísticas.

Avaliação dos Riscos e Benefícios:

Riscos:

Não existem riscos específicos tratando-se de respostas escritas de um breve questionário.

Benefícios:

Validação do método para aplicação na prática clínica de pacientes com paralisia facial.

Comentários e Considerações sobre a Pesquisa:

Adaptação realizada utilizando a técnica de tradução-retradução conforme recomendações da International Society for Pharmaeconimics and Outcome Research, que sugere as seguintes etapas: 1) Preparação: contato com o pesquisador principal que desenvolveu o questionário original para autorização da adaptação e obtido o consentimento da Associação Americana de Fisioterapia detentora dos direitos sobre o artigo original publicado no periódico Physical Therapy. 2) Tradução inicial a partir do original do FDI em Inglês Americano para o português: realizada por dois tradutores independentes cuja primeira língua seja o português. 3) Reconciliação: As versões obtidas são avaliadas por ao menos dois pesquisadores envolvidos no projeto e comparadas para diferenças e submetidas à reconciliação e preparação de uma única versão inicial da tradução para o português. 4) Retradução: Nesta fase a versão inicial em português é então retraduzida para o inglês por outros dois tradutores cuja língua materna seja o inglês americano e que desconhecem o questionário original. 5) Revisão das retraduções: Os pesquisador comparam as retro-traduções com o questionário original e com a versão de reconciliação para determinar se as versões apresentam resultados diferentes do original (perda do significado original), literais (idêntico ao original) ou similar (embora algumas palavras apresentem significados diferentes o conceito do questionário se mantém). 6) Harmonização da retradução: Após as comparações das retraduções modificações são realizadas para conciliar discrepâncias uma segunda versão do questionário é desenvolvida. 7) Desdobramentos cognitivos: A segunda versão do questionário é testada em 20 pacientes para verificar a necessidade de vocábulos alternativos, interpretabilidade, compreensibilidade, e relevância cultural da tradução. O questionário é autoaplicado pelo paciente e após respondê-lo, os pesquisadores solicitam que o paciente registre qual foi o entendimento que o paciente teve de cada item, graduando em pontuação de 1 a 10 (da menor facilidade de entendimento para a maior facilidade) considerando que escores entre 1 e 4 representariam enunciados mal interpretados, entre 5 e 7 um enunciado pouco claro e, entre 8 e 10 um enunciado

claro, conforme proposto por Tavares et al 6. O índice de clareza foi obtido por meio da média das somatórias das notas atribuídas pelos pacientes. Os enunciados que não atingissem o índice 0,4 deveriam ser substituídos, os enunciados que não atingissem o índice 0,8 deveriam ser reformulados e, finalmente, os enunciados que apresentassem o índice final igual ou acima de 0,8 eram considerados adequados quanto ao seu entendimento. 8 – Revisão dos desdobramentos cognitivos e finalização: avaliação da interpretação dos pacientes sobre o questionário traduzido para determinar discrepâncias do significado original pretendido. 9 – Elaboração da versão final: os pesquisadores envolvidos nas etapas anteriores avaliam as modificações obtidas no processo e produzem a versão final traduzida e adaptada para o português falado no Brasil. – Validação do questionário FDI em português e Análise estatística. Aplicação da versão final do questionário FDI em formato teste e reteste em um grupo de 20 pacientes com paralisia facial periférica atendidos no ambulatório de paralisia facial da Disciplina de Otorrinolaringologia do Hospital das Clínicas da UNICAMP.

Considerações sobre os Termos de apresentação obrigatória:
Além do relatório de pesquisa, foi encaminhada a folha de rosto da CONEP assinada pela pesquisadora e complementada por autorização do Coordenador de Assistência do Hospital das Clínicas da Unicamp. Com relação ao TCLE, a linguagem é clara. No TCLE constam o título completo da pesquisa e o nome do pesquisador responsável. Constam a justificativa, uma descrição dos procedimentos envolvidos, riscos e benefícios. Está explicado como será feito o acompanhamento, e que poderá haver esclarecimentos se necessário. Está claro o direito de recusa, e que a recusa não acarretará em consequências para o tratamento do paciente. Está claro que haverá confidencialidade dos dados. Está claro no TCLE que o sujeito irá receber uma via. Há dados do CEP e formas de contato com o pesquisador.

Conclusões ou Pendências e Lista de Inadequações:
Aprovado.

Considerações Finais a critério do CEP:
- O sujeito de pesquisa deve receber uma via do Termo de Consentimento Livre e Esclarecido, na íntegra, por ele assinado.

- O sujeito da pesquisa tem a liberdade de recusar-se a participar ou de retirar seu consentimento em qualquer fase da pesquisa, sem penalização alguma e sem prejuízo ao seu cuidado.

- O pesquisador deve desenvolver a pesquisa conforme delineada no protocolo aprovado. Se o pesquisador considerar a descontinuação do estudo, esta deve ser justificada e somente ser realizada após análise das razões da descontinuidade pelo CEP que o aprovou. O pesquisador deve aguardar o parecer do CEP quanto à descontinuação, exceto quando perceber risco ou dano não previsto ao sujeito participante ou quando constatar a superioridade de uma estratégia diagnóstica ou terapêutica oferecida a um dos grupos da pesquisa, isto é, somente em caso de necessidade de ação imediata com intuito de proteger os participantes.

- O CEP deve ser informado de todos os efeitos adversos ou fatos relevantes que alterem o curso normal do estudo. É papel do pesquisador assegurar medidas imediatas adequadas frente a evento adverso grave ocorrido (mesmo que tenha sido em outro centro) e enviar notificação ao CEP e à Agência Nacional de Vigilância Sanitária – ANVISA – junto com seu posicionamento.

- Eventuais modificações ou emendas ao protocolo devem ser apresentadas ao CEP de forma clara e sucinta, identificando a parte do protocolo a ser modificada e suas justificativas. Em caso de projetos do Grupo I ou II apresentados anteriormente à ANVISA, o pesquisador ou patrocinador deve enviá-las também à mesma, junto com o parecer aprovatório do CEP, para serem juntadas ao protocolo inicial.

- Relatórios parciais e final devem ser apresentados ao CEP, inicialmente seis meses após a data deste parecer de aprovação e ao término do estudo.

-Lembramos que segundo a Resolução 466/2012 , item XI.2 letra e, “cabe ao pesquisador apresentar dados solicitados pelo CEP ou pela CONEP a qualquer momento”.

Este parecer foi elaborado baseado nos documentos abaixo relacionados:

Tipo Documento	Arquivo	Postagem	Autor	Situação
Informações Básicas do Projeto	PB_INFORMAÇÕES_BÁSICAS_DO_PROJETO_594927.pdf	04/10/2015 11:54:40		Aceito
Outros	Anuencia_Oorrino.pdf	04/10/2015 11:54:02	Agnaldo José Graciano	Aceito
Folha de Rosto	Folha_de_Rosto.pdf	04/10/2015 11:53:17	Agnaldo José Graciano	Aceito

Endereço: Rua Tessália Vieira de Camargo, 126
Bairro: Barão Geraldo **CEP:** 13.083-887
UF: SP **Município:** CAMPINAS
Telefone: (19)3521-8936 **Fax:** (19)3521-7187 **E-mail:** cep@fcm.unicamp.br

Outros	Versao_Teste_FDI_Portugues.pdf	04/10/2015 11:02:00	Agnaldo José Graciano	Aceito
Outros	Back_Translation_2_FDI.pdf	04/10/2015 11:00:15	Agnaldo José Graciano	Aceito
Outros	Back_Translation_1_FDI.pdf	04/10/2015 10:59:17	Agnaldo José Graciano	Aceito
Outros	Versao_Recon_FDI.pdf	04/10/2015 10:57:44	Agnaldo José Graciano	Aceito
Outros	Tradutor_2_FDI.pdf	04/10/2015 10:52:50	Agnaldo José Graciano	Aceito
Outros	Tradutor_1_FDI.pdf	04/10/2015 10:51:32	Agnaldo José Graciano	Aceito
Projeto Detalhado / Brochura Investigador	Projeto_Detalhado_FDI.pdf	04/10/2015 10:49:54	Agnaldo José Graciano	Aceito
TCLE / Termos de Assentimento / Justificativa de Ausência	TCLE_FDI.pdf	04/10/2015 10:48:58	Agnaldo José Graciano	Aceito

Situação do Parecer:
Aprovado

Necessita Apreciação da CONEP:
Não

CAMPINAS, 30 de Outubro de 2015

Assinado por:
Renata Maria dos Santos Celeghini
(Coordenador)

Endereço: Rua Tessália Vieira de Camargo, 126
Bairro: Barão Geraldo **CEP:** 13.083-887
UF: SP **Município:** CAMPINAS
Telefone: (19)3521-8936 **Fax:** (19)3521-7187 **E-mail:** cep@fcm.unicamp.br
